# Cisplatin Promotes the Efficacy of Immune Checkpoint Inhibitor Therapy by Inducing Ferroptosis and Activating Neutrophils

**DOI:** 10.3389/fphar.2022.870178

**Published:** 2022-06-13

**Authors:** Ziwei Zhou, Yiming Zhao, Si Chen, Guohui Cui, Wenkui Fu, Shouying Li, Xiaorong Lin, Hai Hu

**Affiliations:** ^1^ Department of Oncology, Sun Yat-Sen Memorial Hospital, Sun Yat-sen University, Guangzhou, China; ^2^ Phase I Clinical Trial Centre, Sun Yat-Sen Memorial Hospital, Sun Yat-sen University, Guangzhou, China; ^3^ Diagnosis and Treatment Center of Breast Diseases, Shantou Central Hospital, Shantou, China

**Keywords:** non–small cell lung cancer, cisplatin, ferroptosis, immune checkpoint inhibitors, neutrophils

## Abstract

The combination of immunotherapy with platinum-based chemotherapy has become the first-line treatment for patients with advanced non–small cell lung cancer (NSCLC) with negative driver gene mutations. However, finding an ideal chemotherapeutic regimen for immunotherapy and exploring the underlying mechanism have noticeably attracted clinicians’ attention. In this study, we found that cisplatin induced ferroptosis of tumor cells, followed by N1 neutrophil polarization in the tumor microenvironment, which in turn remodeled the “cold” tumor to a “hot” one through enhancing T-cell infiltration and Th1 differentiation. Based on the important role of tumor ferroptosis in the immune-promoting effect of cisplatin, we noticed that the combination of a ferroptosis activator showed a synergistic effect with chemoimmunotherapy of epidermal growth factor receptor (EGFR)-mutant NSCLC, which would be an effective strategy to overcome immunotherapy resistance in NSCLC patients harboring driver mutations.

## 1 Introduction

Non–small cell lung cancer (NSCLC) remains the leading cause of cancer-related deaths worldwide (Malvezzi et al., 2012; Apetoh et al., 2015; Global Burden of Disease Cancer et al., 2018). Recent development of immune checkpoint inhibitors (ICIs) has revolutionized the treatment of NSCLC and noticeably improved the outcomes in patients with NSCLC. Despite the successful administration of ICIs, only a subgroup of cancer patients can benefit from immune checkpoint blockade therapy. Some patients do not respond to initial immunotherapy, and the majority of responders eventually develop acquired resistance to ICIs. Accumulating preclinical and clinical evidence indicated that chemotherapy regimens, which are capable of inducing anticancer immunity, can be particularly promising partners for use in combination with ICIs (Hanahan and Weinberg, 2011; Pfirschke et al., 2016; Mathew et al., 2018; Emens et al., 2021). Notably, the combination of a platinum-based doublet and pembrolizumab is the first-line treatment of NSCLC without driver mutations (Langer et al., 2016; Gandhi et al., 2018). However, the underline mechanism of platinum-based chemotherapy on promoting anticancer immune response has not been fully clarified yet. Additionally, epidermal growth factor receptor (EGFR)- or anaplastic lymphoma kinase (ALK)-driven mutations showed an inferior response to immunotherapy. The high frequency of inactive tumor-infiltrating lymphocytes, low tumor mutational load (Rizvi et al., 2015), and weak immunogenicity (Dong et al., 2017) indicate a non-immunogenic or “cold” microenvironment. Thus, exploration of the underlying mechanism of platinum-based chemotherapy on promoting anticancer immune response may lead to the discovery of new solutions to promote ICI outcomes. This knowledge could be significant to developing new combination therapies for patients with NSCLC harboring oncogenic driver mutations and enhance the responses to ICI therapy.

Previous studies indicated that chemotherapeutic agents might induce immunogenic cancer cell death, including pyroptosis (Wang et al., 2017) and ferroptosis (Zhang et al., 2020), with the release of immunostimulating molecules. Research studies revealed that cisplatin could induce different types of cell death, including ferroptosis (Zhang et al., 2020), apoptosis, and pyroptosis. It has been demonstrated that cisplatin-induced pyroptosis plays an important role in enhancing anticancer immune response (Zhang CC. et al., 2019). Ferroptosis is an immunogenic cell death (ICD) which could enhance anticancer immunity in the tumor microenvironment (TME) (Tang et al., 2020). However, the role of ferroptosis in inducing anticancer immune response in NSCLC upon platinum-based chemotherapy has not been evaluated. Herein, we revealed that platinum-based chemotherapy could induce ferroptosis of tumor cells, followed by the activation of anti-tumor neutrophils *in vitro* and *in vivo*, which in turn remodeled the TME through enhancing T-cell infiltration and Th1 differentiation. We also found that a ferroptosis inducer would amplify the anticancer immune response to ICI therapy of NSCLC patients harboring oncogenic driver mutations upon platinum-based chemotherapy.

## 2 Materials and Methods

### 2.1 Data Collection

The transcriptome data of patients with lung adenocarcinoma (LUAD), lung squamous cell carcinoma (LUSC), bladder urothelial carcinoma (BLCA), breast invasive carcinoma (BRCA), esophageal carcinoma (ESCA), and head and neck squamous cell carcinoma (HNSCC) were downloaded from the Cancer Genome Atlas (TCGA) database *via* the Genomic Data Commons (GDC) data portal [Repository (cancer.gov)]. The RNA-seq gene expression level 3 data were then obtained.

### 2.2 Correlation Between Ferroptosis and Immune Scores

The marker gene set of the ferroptosis pathway was obtained from the Kyoto Encyclopedia of Genes and Genomes (KEGG) database (Supplementary Table S3). Single sample gene set enrichment analysis (ssGSEA) was performed to derive the enrichment score, namely, ferroscore in the present study, of the ferroptosis pathway in each sample using an R package called “GSVA” (Hanzelmann et al., 2013). Immune scores were calculated using the ESTIMATE algorithm (Verhaak et al., 2010). The correlation between ferroscores and immune scores in different types of cancer was assessed by Spearman’s correlation analysis.

### 2.3 Analysis of Immune Cells in the Tumor Microenvironment

The tumor-infiltrating immune cells were estimated using the CIBERSORT analytical tool (Newman et al., 2015). The gene expression signatures for 22 immune cells were obtained from the CIBERSORT analytical tool [CIBERSORT (stanford.edu)]. Only cases with a CIBERSORT *p*-value < 0.05 were included for further analysis. The difference in immune cells between high-ferroscore and low-ferroscore patients was analyzed *via* the Mann–Whitney U test. The xCell (Aran et al., 2017), a robust computational method that converts bulk transcriptomes to enrichment scores of 64 immune and stromal cells, was also applied to verify the conclusions.

### 2.4 Gene Ontology and Kyoto Encyclopedia of Genes and Genomes Pathway Enrichment Analysis

Patients were divided into two groups, namely, the Ferroscore-H (high ferroscore) group and the Ferroscore-L (low ferroscore) group, and were compared in terms of the median level of the ferroscore. The “limma” package in R software was used to identify differentially expressed genes (DEGs) in the two groups (Ritchie et al., 2015). A false discovery rate (FDR) < 0.05 combined with |log2 (fold change) | >1 was set as the threshold for the identification of DEGs. All DEGs were analyzed by the GO and KEGG pathway enrichment analyses. An FDR *p*-value <0.05 was considered statistically significant for the enrichment analysis.

### 2.5 Gene Set Enrichment Analysis

Gene set enrichment analysis (GSEA) was performed in the ferroscore-H and ferroscore-L groups using the “ClusterProlier” package in R software (Subramanian et al., 2005; Zhang M. et al., 2019). The GO and KEGG pathways were analyzed using this method. Enriched gene sets with a nominal *p*-value < 0.05 and FDR q < 0.05 were considered to be enrichment significant.

### 2.6 Cells and Cell Culture

A549 cells and LLC cells from the American Type Culture Collection (ATCC, Manassas, VA, United States) were cultured in a Dulbecco’s modified Eagle’s medium (DMEM) supplemented with 10% fetal bovine serum (FBS). PC9 cells were maintained in a Roswell Park Memorial Institute (RPMI)-1640 medium supplemented with 10% FBS. Human neutrophils were isolated from the peripheral blood of healthy donors using density gradient centrifugation *via* Percoll (Pharmacia Fine Chemicals, Uppsala, Sweden). Total immune cells are obtained from peripheral blood by lysis of red blood cells. Neutrophils and total immune cells were cultured in a DMEM with 10% FBS.

### 2.7 Drugs

The chemicals used were as follows: cisplatin (S1166; Selleck Chemicals, Houston, TX, United States), ferrostatin-1-1 (S7243, Selleck), and RSL3 (S8155, Selleck).

### 2.8 Quantitative Reverse Transcription Polymerase Chain Reaction

Total RNA was extracted using TRIzol reagent (Invitrogen, Carlsbad, CA, United States) and was reversely transcribed into cDNA using a PrimeScript RT Reagent kit (TaKaRa, Tokyo, Japan). The RT-qPCR was performed using a SYBR Premix Ex Taq kit (TaKaRa) according to the manufacturer’s instructions. Data were collected and analyzed using LightCycler 480 software (Roche, Basel, Switzerland).

### 2.9 Cytotoxicity Assay

Calcein-AM–labeled A549 cells or PC9 cells were co-cultured with neutrophils or total immune cells for 8 h. The effector-to-target ratio was 5:1. Co-cultured cells were maintained in 24-well plates. Mixed cells of co-culture system were dyed with 7-AA-D for cell death rate analysis. The death rate of tumor cells was assessed by flow cytometry. The dead tumor cells were considered as calcein and 7-AA-D double-positive cells.

### 2.10 Lipid Peroxidation Assay

Lipid peroxidation assay was carried out using a lipid peroxidation kit (ab243377; Abcam, Cambridge, United Kingdom) according to the manufacturer’s instructions. Briefly, a lipid peroxidation sensor was added to the tumor cell culture medium and incubated for 30 min. After washing with phosphate-buffered saline (PBS), fluorescence was detected in the fluorescein Isothiocyanate (FITC) channel and P-phycoerythrin (PE) channel by flow cytometry. The lipid peroxidation was quantified as the ratio of PE (non-oxidized)/FITC (oxidized)-positive cell count.

### 2.11 Magnetic Activated Cell Sorting of CD4^+^ T Cells

Human PBMCs were isolated from the peripheral blood of healthy donors using Ficoll density gradient centrifugation. CD4^+^ T helper cells were isolated from PBMCs using CD4 microbeads (130-045-101; Miltenyi, Bergisch Gladbach, Germany), a LS Magnetic Column (Miltenyi), and a MiniMACS separator.

### 2.12 Immunohistochemistry

The IHC staining was performed using Ly-6G (Santa Cruz Biotechnology, Inc., Dallas, TX, United States) and T-bet (Proteintech, Rosemont, IL, United States) antibodies according to standard protocols on formalin-fixed and paraffin-embedded tumor tissues. Immunolabeling was visualized with a mixture of 3,3′-diaminobenzidine (DAB) solution (ZSGB-BIO, Beijing, China), followed by counterstaining with hematoxylin.

### 2.13 Animal Experiments

6-week-old C57BL/6 mice were purchased from Beijing Vital River Laboratory Animal Technology Co., Ltd. (Beijing, China). Mice were kept in a specific pathogen-free (SPF) animal house at 28°C with 50% humidity. 7 × 106 LLC cells were inoculated into the subcutaneous layer of 6-week-old female C57BL/6 mice (*n* = 6 mice per group). After the xenografts became palpable, mice were injected with 10 mg/kg cisplatin (DDP) or 10 mg/kg DDP plus 10 mg/kg ferrostatin-1-1 intraperitoneally. Anti-PD1 antibody was injected intraperitoneally at a dose of 8 mg/kg 3 days later. After 9 days of observation, the mice were killed. Tumors and harvested organs were subjected to IHC staining.

### 2.14 Primary Cell Isolation

Cut tumors and get rid of the peripheral tissue. Put the tumor tissue into PBS and wash several times. Use the scalpel to chop the tissue for 5 min. Put the minced tissue into a 50-ml centrifuge tube and add 10 ml/g tissue of DMEM with 300 units/ml collagenase IV and 100 units/ml hyaluronidase for 1 h of shaking at 200 rpm/37°C. Centrifuge at 600 g for 10 min. Re-suspend with 10 ml washing buffer every time until the supernatant is clean and the pellet is white. Wash with DMEM once and re-suspend in 2 ml pre-warmed trypsin. Put into a 35-mm culture dish and incubate at 37°C for 20 min. Gently pipet the solution to mix every 5 min. Add the medium into neutralized trypsin and centrifuge at 1,000 g for 3 min. Re-suspend the pellet in 1–2 ml pre-warmed washing buffer with 5 mg/ml dispase and 0.1 mg/ml DNase I. Gently pipet the solution to mix every 5 min. Add the medium into neutralized trypsin and centrifuge at 1,000 g for 3 min. Re-suspend the pellet in 1–2 ml pre-warmed washing buffer with 5 mg/ml dispase and 0.1 mg/ml DNase I. Gently pipet the solution to mix and incubate at room temperature for 2 min. Briefly centrifuge to achieve the fibers down and then filter through a 70-μm cell strainer to obtain single cell suspension. Centrifuge at 1,000 g for 3 min and re-suspend the cells in a growth medium.

### 2.15 Enzyme-Linked Immunosorbent Assay

ELISA was performed using human interferon gamma (IFN-γ) (430107; BioLegend, San Diego, CA, United States), human C-X-C Motif Chemokine Ligand 1 (CXCL1) (RAB0116; Sigma-Aldrich, St. Louis, MO, United States), and human C-X-C motif chemokine ligand 2 (CXCL2) (DY276-05; R&D Systems, Minneapolis, MN, United States), according to the manufacturers’ protocols.

## 3 Results

### 3.1 Cisplatin Promoted Tumor Ferroptosis That Was Correlated With Immune Score

**FIGURE 1:**
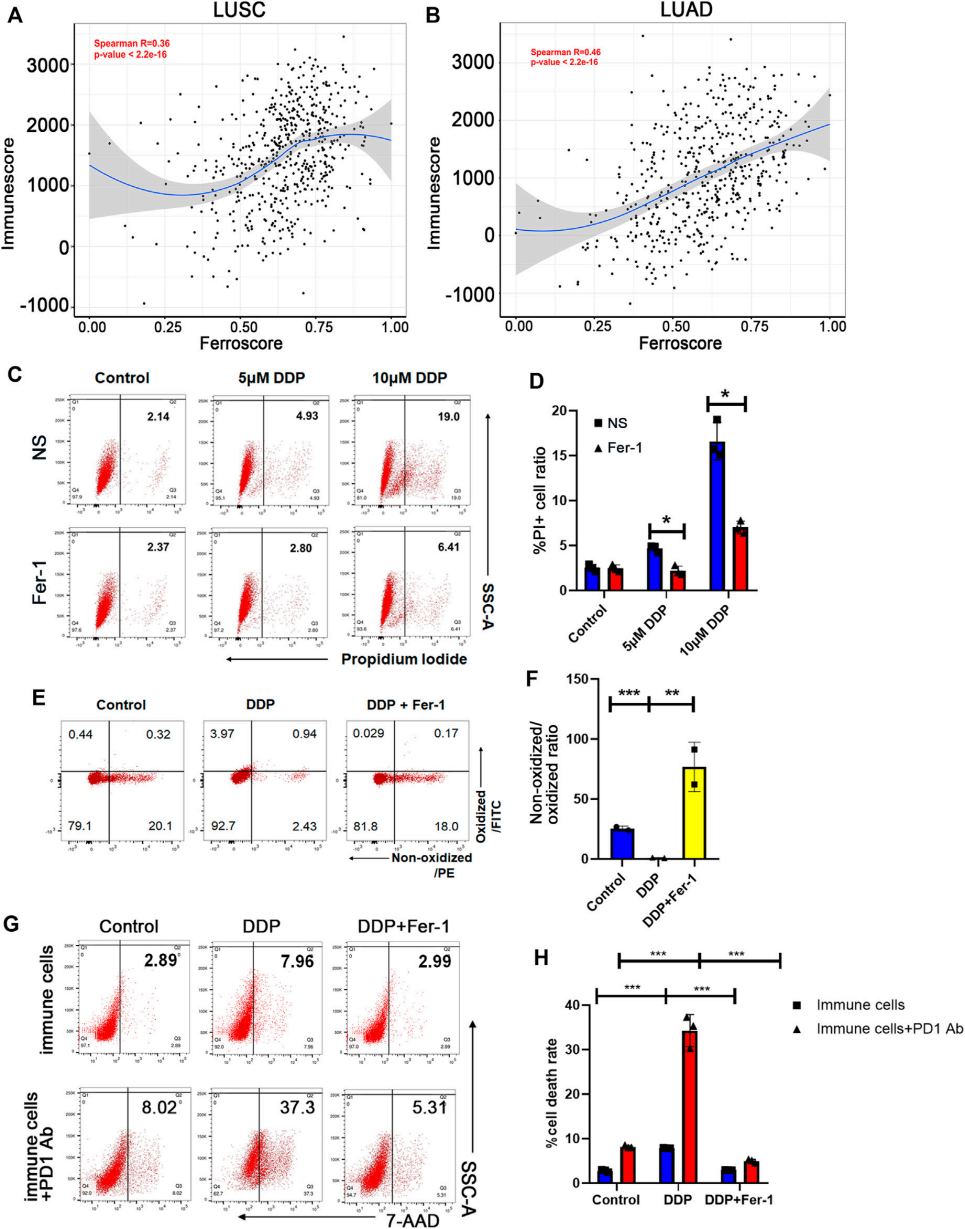
Cisplatin promoted tumor ferroptosis that was correlated with immune score. **(A)** and **(B)** The ferroscore correlated with immunescore in LUSC **(A)** and LUAD **(B)** cohorts of TCGA database. **(C)** Cell death ratio of A549 cells treated with 5 μM cisplatin (DDP) or 10 μM DDP for 8 h measured by flow cytometry (NS: normal saline). **(D)** Quantification of data in **(C)**. **(E)** Lipid peroxidation of A549 cells treated with 10 μM DDP or rescued by 10 μM ferrostatin-1 (Fer-1) for 8 h determined by flow cytometry. **(F)** Quantification of data in **(E)**. The ratio of non-oxidized lipid (PE)/oxidized lipid (FITC) fluorescence intensities decreases in DDP-treated cells, indicating that DDP induces lipid peroxidation, while ferrostatin-1 rescued this effect. **(G)** Cell death ratio of A549 cells co-cultured with immune cells before or after anti-PD1 antibody treatment, determined by flow cytometry. The immune cells were pretreated with supernatant of A549 cells, which were given DDP or DDP plus ferrostatin-1. **(H)** Quantification of data in **(G)**. DDP could increase cytotoxic effect of immune cells and anti-PD1 antibody efficacy, which would be rescued by ferrostatin-1. Bar graphs represent the mean ± SD of indicated samples. ∗*p* < 0.05, ∗∗*p* < 0.01, ∗∗∗*p* < 0.001.

To explore whether cisplatin could trigger ferroptosis to enhance anticancer immune response in NSCLC treatment, we first attempted to indicate whether ferroptosis could be correlated with the tumor immune microenvironment (TIME) of NSCLC using TCGA database. The ferroptosis status was defined as “high” or “low” ferroscore according to the expressions of ferroptosis-related genes *via* the KEGG pathway. The TIME status was defined as an immune score featured with different types of immune cells that infiltrated tumor tissues (Verhaak et al., 2010). We revealed that the ferroscore was significantly correlated with immune score both in the LUSC and LUAD cohorts from TCGA database (Figures 1A,B). Furthermore, the strong correlation between ferroptosis and enhanced immune cell infiltration was not limited to NSCLC. We found a significant correlation between ferroscore and immune score among different types of cancer, including BLCA, BRCA, ESCA, and HNSCC cohorts in TCGA database (Supplementary Figures S1A–D). Cisplatin in combination with immunotherapy is an important treatment option in these tumors. This finding is consistent with our assumption that ferroptosis could act as an enhancement factor to promote the anticancer immune response.

Distinct from other cell death modalities, ferroptosis is marked by iron-dependent lipid peroxidation (Hassannia et al., 2019). In order to indicate whether cisplatin could induce ferroptosis of NSCLC cells, we detected cell death and lipid peroxidation of A549 lung cancer cells upon cisplatin treatment. Our results showed that cisplatin induced A549 cell death in a dose-dependent manner, and about 50% of cell death could be rescued by ferrostatin-1, as a ferroptosis inhibitor (Figures 1C,D). In addition, cisplatin also mediated lipid peroxidation and could be rescued by ferrostatin-1 (Figures 1E,F), indicating that cisplatin could induce ferroptosis of NSCLC cells. Ferroptosis has been reported to enhance the anticancer immune response (Tang et al., 2020). Then, we examined whether cisplatin-mediated ferroptosis could enhance anti-tumor effect of immune cells and play a synergistic role with ICIs in NSCLC treatment. The supernatants of A549 cells that have been treated with cisplatin or cisplatin + ferrostatin-1 were collected. Immune cells, obtained from peripheral blood after red cell lysis, were pretreated with the supernatants of A549 cells for 24 h. Then, the anti-tumor cytotoxic effect of immune cells was examined by co-culture of A549 cells with these immune cells. Flow cytometry assay was used to assess the cell death ratio of A549 cells, and it was revealed that immune cells pretreated with supernatant of cisplatin-treated NSCLC cells had a significantly stronger cytotoxic effect, whereas the supernatant of cisplatin + ferrostatin-1–treated NSCLC cells could not significantly boost the anti-tumor effect of immune cells (Figures 1G,H). Furthermore, immune cells exhibited a stronger anti-tumor ability when pretreatment with programmed cell death protein 1 (PD-1) antibody was performed in the co-culture system (Figures 1G,H). The findings mentioned above suggested that cisplatin could induce ferroptosis of NSCLC cells, thereby promoting the anti-tumor cytotoxic ability of immune cells to augment the therapeutic effect of ICIs.

### 3.2 Neutrophil Activation Pathway Was Significantly Upregulated in the High Ferroscore Cohort

**FIGURE 2:**
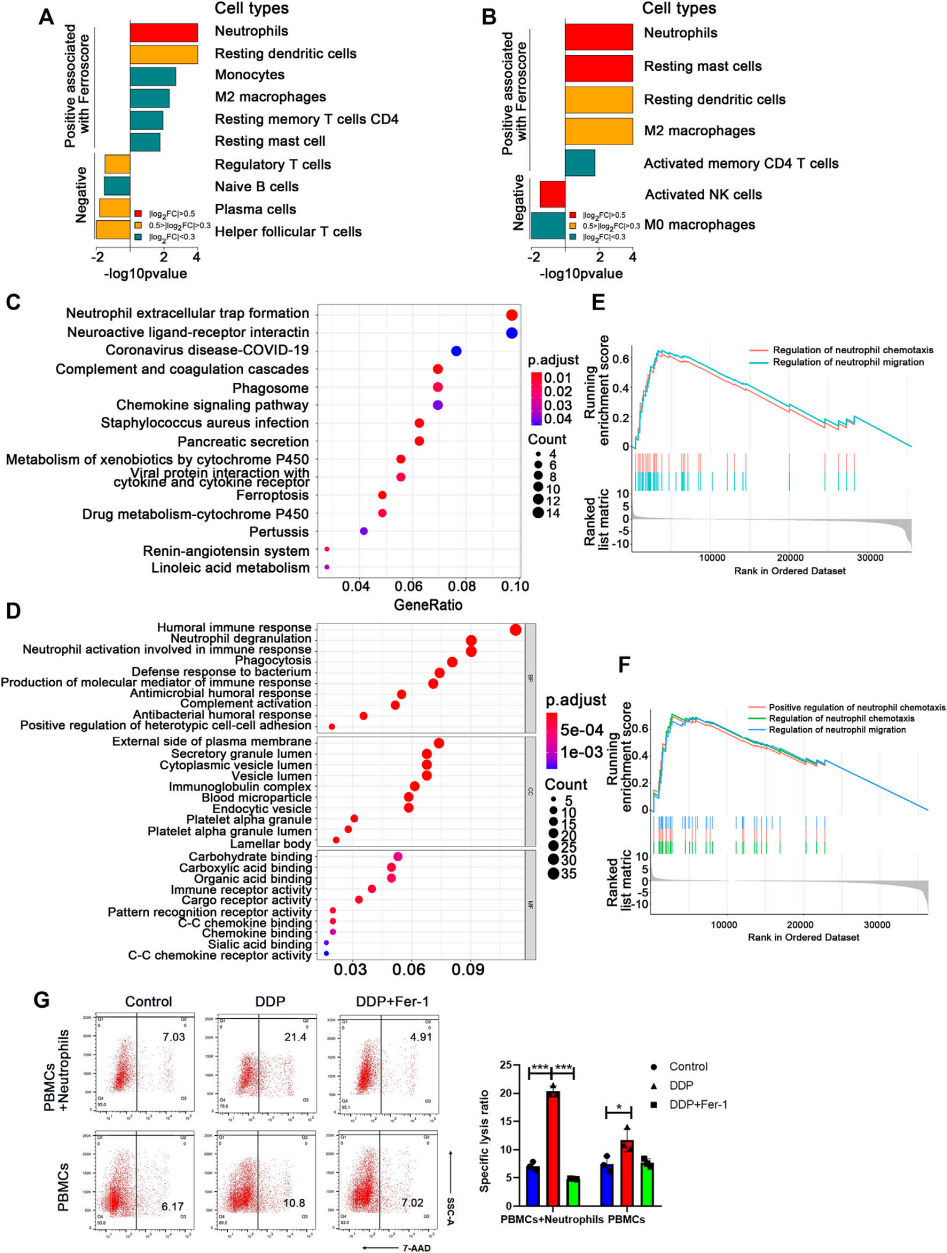
Neutrophil activation pathway was significantly upregulated in the high ferroscore cohort. **(A)** and **(B)** CIBERSORT algorithms analysis showed the cell types, positive and negative, associated with ferroscore in LUSC **(A)** and LUAD **(B)** cohorts (FC: fold change). **(C)** and **(D)** Kyoto Encyclopedia of Genes and Genomes (KEGG) pathway and gene ontology (GO) enrichment analysis of differentially expressed genes between high- and low-ferroscore samples. **(E)** and **(F)** Upregulation of neutrophil migration or chemotaxis-related signature genes in high ferroscore samples relative to low ferroscore samples by gene set enrichment analysis (GSEA) in both LUSC **(E)** and LUAD **(F)** cohort. **(G)** In the presence of neutrophils, DDP pretreatment significantly increased the A549 death ratio, which could be restored by Fer-1. However, DDP pretreatment failed to induce obvious A549 cell death ratio in the absence of neutrophils. ∗*p* < 0.05, ∗∗∗*p* < 0.001.

To figure out which type of the immune cells could be involved in the ferroptosis-induced anti-tumor immune response, the infiltration levels of various types of immune cells in the tumor tissues from TCGA database were calculated using the CIBERSORT analytical tool (Newman et al., 2015). We found that neutrophils, resting mast cells, resting dendritic cells, and M2 macrophages were significantly more abundant in the ferroscore-H (high ferroscore) group in both LUSC and LUAD cohorts (Figures 2A,B) than in the ferroscore-L (ferroscore low) group. Among these immune cells, neutrophils showed the most prominent enrichment in the ferroscore-H group in both LUSC and LUAD cohorts. Consistently, xCell algorithm (Aran et al., 2017) also demonstrated that the ferroscore-H group was correlated with a higher level of neutrophils (Supplementary Figure S2). Moreover, the KEGG pathway and Gene Ontology enrichment analyses of the differentially expressed genes (DEGs) revealed that neutrophil-related pathways, such as neutrophil extracellular trap formation, neutrophil degranulation, and morphological and behavioral changes of neutrophils after being stimulated, were the most activated pathways among the top-ranking pathways in the ferroscore-H group in the LUSC TCGA dataset (Figures 2C,D). Furthermore, gene set enrichment analysis (GSEA) was used to examine the correlation between the ferroscore and neutrophil-related pathways. Regulation of neutrophil chemotaxis and migration pathways were strongly correlated with high ferroscore (Figures 2E,F). Neutrophils are the most frequent type of innate immune cells and are the first cells to arrive at sites of developing inflammation. It has been shown that neutrophils are often the first cells to arrive both at a wound and during the early initiation phases of carcinogenesis (Feng et al., 2012). Once neutrophils infiltrate tumor tissues, they may undergo polarization to switch to N1/anti-tumor or N2/pro-tumor phenotype induced by the TME (Giese et al., 2019). Thus, we hypothesized that the N1-polarized neutrophils in these tumor tissues could play a key role in the ferroptosis-induced anti-tumor immune response.

Based on the above bioinformatics analysis, we initially put forward the hypothesis that neutrophils play a key role in the anti-tumor immune response induced by ferroptosis. Then we constructed a co-culture system to verify the above hypothesis. The tumor cells pretreated with DDP or DDP + Fer-1 were co-cultured with immune cells (PBMCs + neutrophils) or PBMCs for 12 h, respectively. The tumor death ratio was detected by flow cytometry (Figure 2G). The results suggest that the death rate of tumor cells pretreated with DDP is 2 times more than that in the control group with the presence of neutrophils, while Fer-1 can restore the death rate of tumor cells after being attacked by immune cells. However, the death rate of tumor cells did not show obvious change, whether they were pretreated with DDP or DDP + Fer-1 in the co-culture system without neutrophils. The above results indicated that neutrophils play an important role in anti-tumor immune response.

### 3.3 N1-Polarized Neutrophils Induced by Cisplatin-Mediated Tumor Ferroptosis Exerted an Anti-Tumor Effect

**FIGURE 3:**
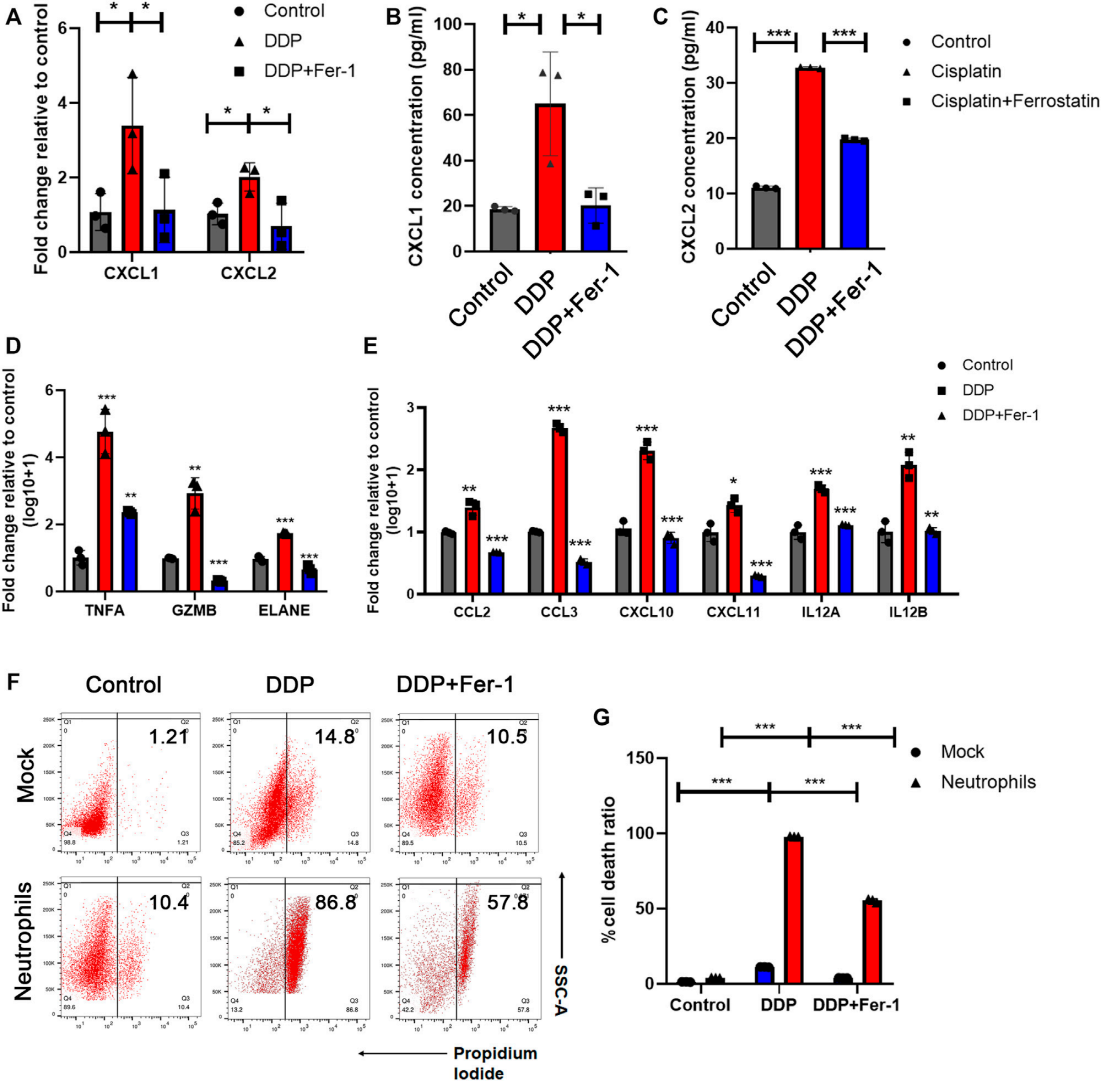
N1-polarized neutrophils induced by cisplatin-mediated tumor ferroptosis exerted an anti-tumor effect. **(A)** Relative mRNA expression of CXCL1 and CXCL2 was tested by RT-qPCR assay in A549 cells treated with 5 μM DDP or rescued by 10 μM ferrostatin-1. **(B)** and **(C)** Concentration of CXCL1 **(B)** and CXCL2 **(C)** released by A549 cells in culture medium, which was tested by ELISA assay. A549 cell were treated with 5 μM DDP or rescued by 10 μM ferrostatin-1. **(D)** and **(E)** Relative mRNA expression of neutrophil cytotoxic markers **(D)** and pro-inflammatory markers **(E)** were tested by RT-qPCR assays in neutrophils co-cultured with A549 cells, which were pretreated with 5 μM DDP or rescued by ferrostatin-1. **(F)** Cell death ratio of A549 cells treated with DDP or rescued by ferrostatin-1 before and after being co-cultured with neutrophils. **(G)** Quantification of data in **(F)**. DDP pretreatment remarkably increases the cytotoxic effect of neutrophils which would be rescued by ferrostatin-1. Bar graphs represent the mean ± SD of indicated samples. ∗*p* < 0.05, ∗∗*p* < 0.01, ∗∗∗*p* < 0.001.

According to the analysis above, we further supposed that cisplatin-based chemotherapy could mediate tumor cell ferroptosis-induced neutrophil infiltration and N1 neutrophil polarization and, thus, enhanced the anti-tumor immune response. In order to validate this assumption, the cytokines for neutrophil recruitment, including CXCL1 and CXCL2 (De Filippo et al., 2013; Paudel et al., 2019), were examined in the cisplatin-treated tumor cells. The RT-qPCR (Figure 3A) and ELISA (Figures 3B,C) showed that cisplatin-treated A549 cells have higher expression levels of CXCL1 and CXCL2 than the cells without treatment. In addition, the increased RNA and cytokine levels of CXCL1 and CXCL2 could be restored by ferrostatin-1 treatment, suggesting that cisplatin enhanced the expression levels of CXCL1 and CXCL2 expression *via* inducing ferroptosis of tumor cells (Figures 3A–C). These findings supported the idea that cisplatin-mediated tumor cell ferroptosis could recruit neutrophils *via* promoting the expression levels of CXCL1 and CXCL2 in tumor cells.

It has been reported that neutrophils in tumor tissue exhibited an N1/pro-inflammatory phenotype and function as anticancer immune cells (Giese et al., 2019). N1 neutrophils could directly play an anti-tumor role by modulating the expression levels of cytotoxic effectors, including tumor necrosis factor-α (TNF-α) (Balkwill, 2009), granzyme B (GZMB) (Martin et al., 2018), and elastase (ELANE) (Cui et al., 2021). Additionally, N1 neutrophils could be associated with the anti-tumor effects of other immune cells by secreting pro-inflammatory cytokines, such as C-C Motif Chemokine Ligand 2 (CCL2) (Nagarsheth et al., 2017), C-C Motif Chemokine Ligand 3 (CCL3) (Chheda et al., 2016), C-X-C Motif Chemokine Ligand 10 (CXCL10) (Chheda et al., 2016), C-X-C Motif Chemokine Ligand 11 (CXCL11) (Chheda et al., 2016), and interleukin-12 (IL12) (Vignali and Kuchroo, 2012). We attempted to indicate whether cisplatin-mediated tumor cell ferroptosis could induce the anti-tumor pro-inflammatory phenotype of neutrophils. A549 cells were first treated with cisplatin with or without ferrostatin-1 for 8 h. Then, the culture medium was replaced with a fresh medium, and neutrophils were added into the co-culture system for 12 h. We found that cisplatin-pretreated tumor cells stimulate neutrophils which expressed higher levels of cytotoxic effectors, including TNF-α, GZMB, and ELANE (Figure 3D), and pro-inflammatory cytokines, such as CCL2, CCL3, CXCL10, CXCL11, and IL12 (Figure 3E). However, when tumor cells were pretreated with cisplatin + ferrostatin-1, they could not effectively stimulate neutrophils to express these cytotoxic effectors or pro-inflammatory cytokines (Figures 3D,E).

As N1 neutrophils could exert a direct cytotoxic effect on tumor cells (Cui et al., 2021), the cytotoxicity of neutrophils was evaluated by the flow cytometry assay. A549 cells treated with cisplatin showed a moderate cell death, and the presence of ferrostatin-1 partially rescued the cell death (Figures 3F,G). When neutrophils were added into the culture system, an overwhelming amount of tumor cell death was observed in the cisplatin treatment group, which could be partially rescued by the presence of ferrostatin-1 (Figures 3F,G). The results indicated that cisplatin-mediated tumor cell ferroptosis could recruit neutrophils and boost the pro-inflammatory effects of neutrophils.

### 3.4 N1-Polarized Neutrophils Induced by Tumor Ferroptosis Promoted T Cell Infiltration and Th1 Differentiation

**FIGURE 4:**
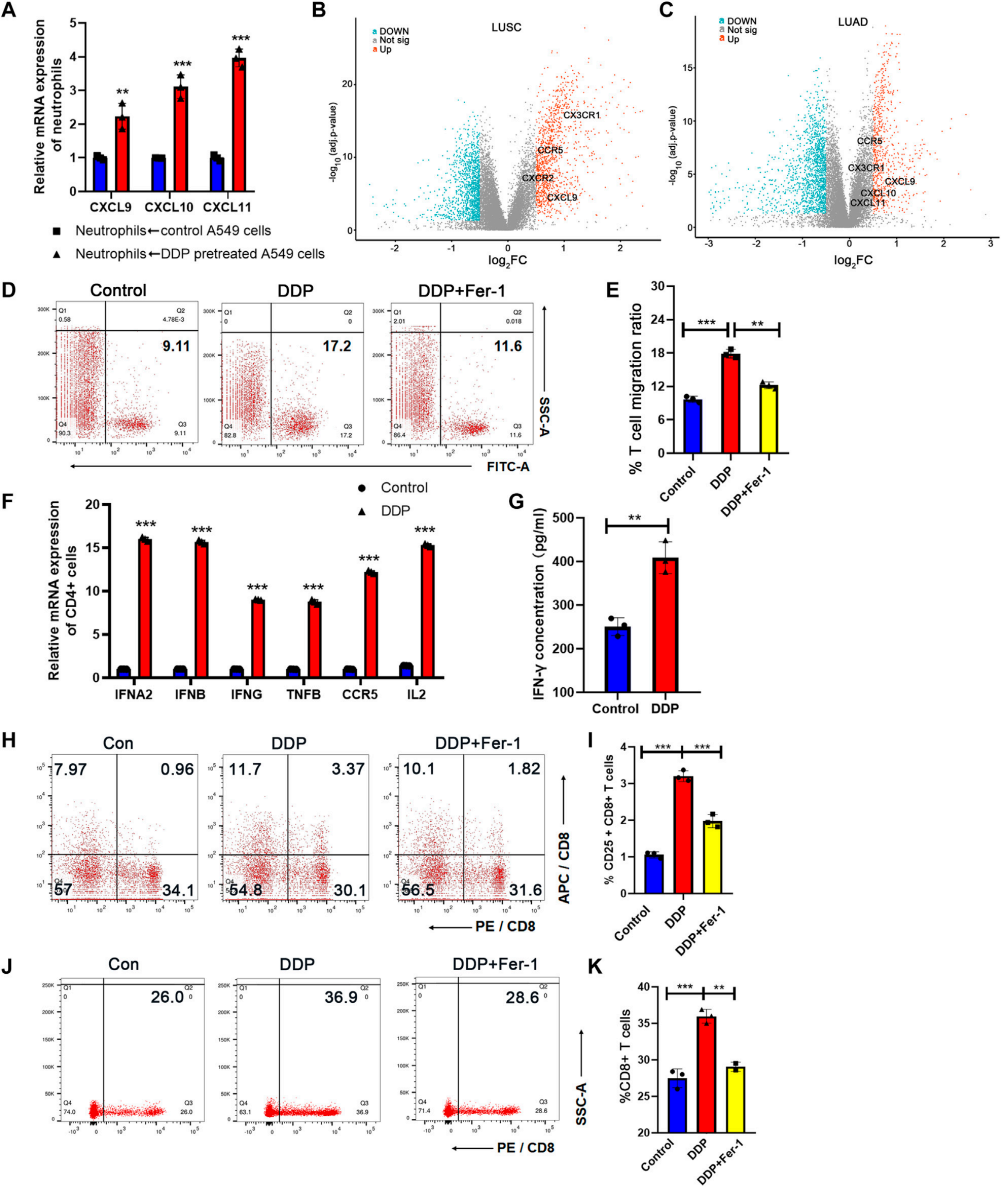
N1-polarized neutrophils induced by tumor ferroptosis promoted T-cell infiltration and Th1 differentiation. **(A)** CXCL9, CXCL10, and CXCL11 mRNA expression levels of neutrophils increase after being co-cultured with DDP-pretreated A549 cells, as measured by RT-qPCR. **(B)** and **(C)** Volcano plot between high- and low-ferroscore groups in LUSC **(B)** and LUAD **(C)** cohorts. Chemokines for T-cell infiltration was highlighted in the plot. **(D)** T-cell migration ratio measured by flow cytometry after being co-cultured in the transwell system with neutrophils. The neutrophils were activated by A549 cells, pretreated with DDP or rescued by ferrostatin-1. **(E)** Quantification of data in **(D)**. **(F)** Markers of Th1 subtypes of CD4-positive T cells were determined by RT-qPCR. The CD4-positive T cells were co-cultured with neutrophils, which are making contact with cisplatin-pretreated A549 cells or ferrostatin-1–rescued A549 cells. **(G)** IFN-γ concentration of CD4-positive T-cell culture medium increased after being co-cultured with neutrophils in the transwell system, measured by ELISA assay. The neutrophils are contacted with cisplatin-pretreated A549 cells or controlled A549 cells. Bar graphs represent the mean ± SD of indicated samples. **(H)** CD8^+^ T-cell activation measured by flow cytometry after being co-cultured by N1 neutrophils or control neutrophils. **(I)** Quantification of data in **(H)**. **(J)** The proportion of CD8 T cells in the migrated lymphocytes was measured by flow cytometry. **(K)** Quantification of data in **(J)**. ∗*p* < 0.05, ∗∗*p* < 0.01, ∗∗∗*p* < 0.001.

We found that cisplatin-mediated tumor cell ferroptosis could stimulate the N1-polarized neutrophils. N1-polarized neutrophils could not only directly kill tumor cells but also remodel the TME through pro-inflammatory signals (Eruslanov et al., 2014). We determined whether tumor cell ferroptosis-induced N1-polarized neutrophils could express cytokines to activate T cells for anti-tumor immune response. Chemokines associated with T-cell infiltration were tested on neutrophils that were co-cultured with A549 cells. We found that when neutrophils were co-cultured with cisplatin-pretreated tumor cells, they expressed significantly higher levels of chemokines, such as CXCL9, CXCL10, and CXCL11 (Figure 4A), which could be key regulators of recruitment of T cells into tumor tissues (Muller et al., 2010). Consistently, in the ferroscore-H group from TCGA dataset, high expression levels of CX3CR1, CCR5, CXCL9, CXCL10, and CXCL11 were detected (Figures 4B,C, Supplementary Tables S1, S2), which indicated a higher T-cell infiltration in the TME. Next, the ability of neutrophils to enhance T-cell infiltration was evaluated. A549 cells were pretreated with DDP and then co-cultured with neutrophils. The neutrophils were collected from the co-culture system and seeded into the lower chamber of the transwell system. Calcium-dyed T cells were then seeded into the upper chamber. After 8 h, T cells that moved into the lower chamber were analyzed by flow cytometry, and it was revealed that neutrophils co-cultured with cisplatin-pretreated A549 cells had a stronger potential for T-cell infiltration (Figures 4D,E).

In addition to cytokines for T-cell infiltration, we noticed that the levels of IL12A and IL12B were also upregulated in neutrophils that were co-cultured with cisplatin-pretreated A549 cells (Figure 3E). IL12A and IL12B are key inducers for the differentiation of naive T cells from Th1 cells (Hsieh et al., 1993). Th1 cells are a subset of helper T cells that have a strong potential to evoke cell-mediated immune response in the TME (Huang et al., 2021). Th1 cell emerged as a new therapeutic target to enhance T-cell infiltration and turning “cold” tumors into “hot” ones. We co-cultured CD4^+^ T cells sorted by immunomagnetic beads from peripheral blood mononuclear cells (PBMCs) with neutrophils, which were stimulated by ferroptosis A549 cells. RT-qPCR assays demonstrated that CD4+T cells expressed elevated levels of interferon alpha 2 (IFNA2), interferon beta (IFNB), interferon gamma (IFNG), tumor necrosis factor B (TNFB), C-C Motif chemokine receptor 5 (CCR5), and interleukin 2 (IL2) when they were co-cultured with neutrophils that had been co-cultured with cisplatin-pretreated A549 cells (Figure 4F). ELISA assay indicated that CD4^+^ T cells co-cultured with stimulated neutrophils had more IFN-γ secretion (Figure 4G), supporting a Th1 differentiation of these T cells. The results mentioned above indicated that cisplatin-treated NSCLC cells could stimulate N1-polarized neutrophils and thus enhanced the T-cell infiltration and Th1 differentiation in the TME to boost the anti-tumor immune response.

Cytotoxic CD8 T lymphocytes (CTL cells) are the most direct and important cell subtype for anti-tumor immunity. Many studies show that the responsiveness of CD8 T cells to tumor cells can be enhanced by neutrophils (Governa et al., 2017; Mysore et al., 2021). Moreover we have revealed that N1 neutrophils have a significant promoting effect on Th1 cells, which play an important role in promoting CTL activation, proliferation, and differentiation into memory T cells (Borst et al., 2018). Therefore, we tested the activation and migration ability of CD8 cells when co-cultured with neutrophils. We constructed a co-culture system of neutrophils and PBMC cells according to the previous method. First, activated neutrophils were placed in the lower transwell chamber, and PBMCs were placed in the upper transwell chamber (pore size of 1 μm, to prevent PBMC from entering the lower chamber). CD25 is an important cell surface marker for T-cell activation. After 24 h of co-culture, the proportion of CD8 and CD25 double-positive cells in the upper chamber PBMCs was detected by flow cytometry (Figures 4H,I). The results suggested that N1 neutrophiles polarized by tumor cells pretreated with DDP significantly promoted the activation of CD8 cells, while Fer-1 could restore its effect. Next, we used a transwell chamber with a pore size of 5 μm to perform migration assay of CD8 T cells. N1 neutrophiles were placed in the lower chamber. PBMCs were placed in the upper chamber. After 36 h, we used flow cytometry to detect the percentage of CD8 cells in the migrated lymphocytes that entered the lower chamber (Figures 4J,K). The results suggested that the percentage of CD8 cells was significantly higher after being attracted by N1 neutrophiles. The above results verify that N1 neutrophils promote the anti-tumor activity of CTLs.

### 3.5 Cisplatin-Induced Ferroptosis Promoted Efficacy of Immune Checkpoint Inhibitor Therapy *in Vivo*

**FIGURE 5:**
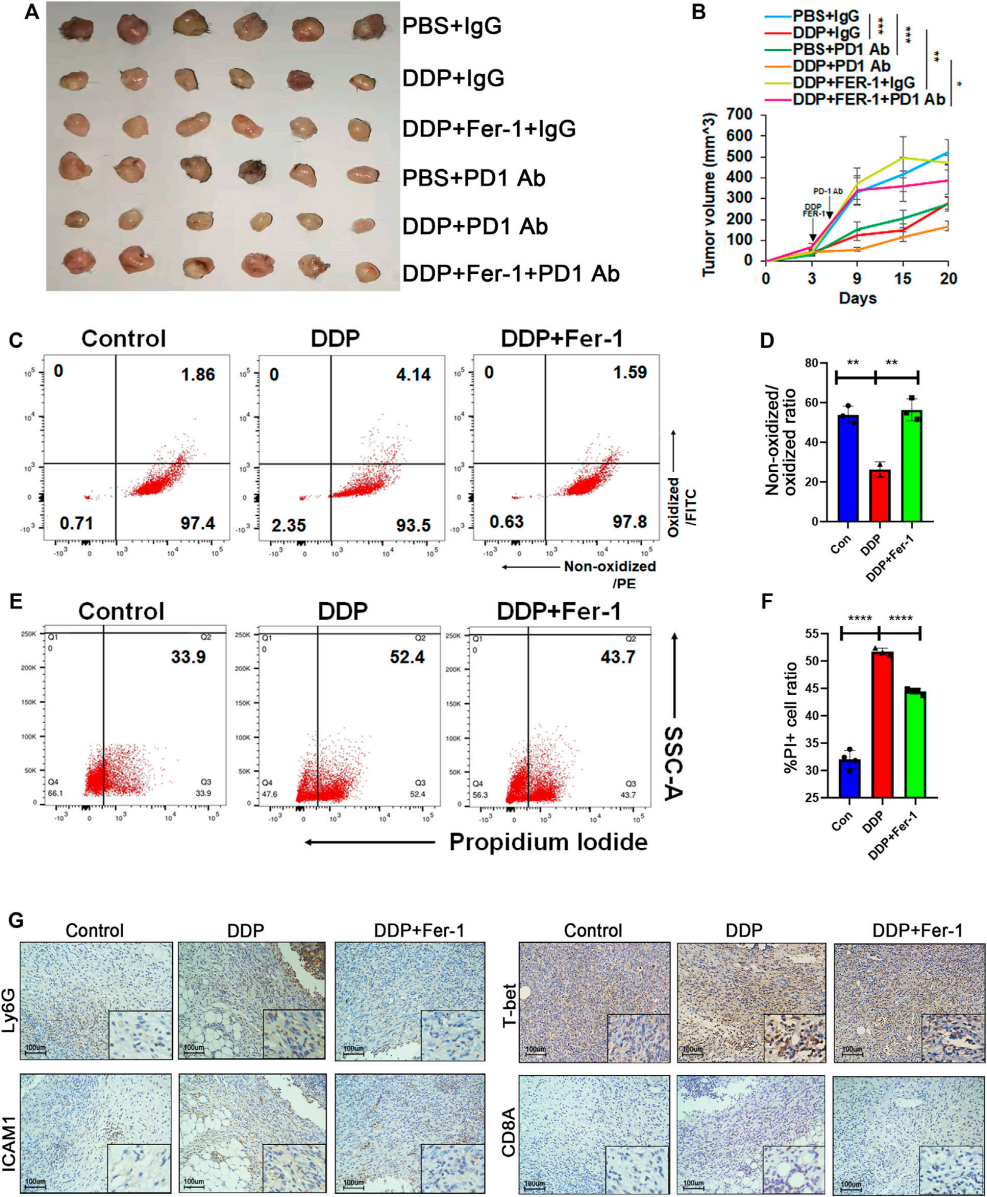
Cisplatin-induced ferroptosis promoted efficacy of ICI therapy *in vivo.* **(A)** and **(B)** Tumor image **(A)** and tumor growth **(B)** of Lewis lung cancer cell (LLC)-bearing C57 mice. When the tumor size was palpable, LLC xenografts were injected with DDP intraperitoneally (5 mg/kg once) and then treated with ferrostatin-1 (10 mg/kg once) or PBS on the same day. Anti-PD1 antibody was injected intraperitoneally the day after DDP or DDP plus ferrostatin-1 administration. Xenografts were harvested 18 days post injection. **(C)** and **(D)** Primary cells were isolated from xenografts 3 days after DDP or DDP plus ferrostatin-1 administration. DDP treatment group showed increased lipid peroxidation while ferrostatin-1 rescued this effect. **(E)** and **(F)** PI staining of primary cells isolated from xenografts indicated that DDP increased cell death ratio while ferrostatin-1 rescued this effect. **(G)** Representative immunohistochemical images of paraffin-embedded xenograft sections. The DDP treatment group showed increased Ly6G and ICAM1 level, which indicated N1 neutrophils. T-bet and CD8A, representing Th1 cells and CD8 T cells, respectively, were also upregulated. Bar graphs represent the mean ± SD of indicated samples. ∗*p* < 0.05, ∗∗*p* < 0.01, ∗∗∗*p* < 0.001.

Clinical trials have shown that different types of lung cancer could benefit from ICI therapy combined with cisplatin-based chemotherapy (Paz-Ares et al., 2020; Rodriguez-Abreu et al., 2021). In order to indicate whether cisplatin-mediated ferroptosis could be involved in synergistic effect of ICI therapy combined with chemotherapy, we attempted to determine whether a ferroptosis inhibitor (ferrostatin-1) could hamper the synergistic efficacy of ICI therapy plus cisplatin in the treatment of Lewis lung carcinoma (LLC) cell lines. C57BL/6 mice were used to inoculate LLC cells. After the tumor cells became palpable, 10 mg/kg cisplatin and 10 mg/kg ferrostatin-1 were administrated intraperitoneally. Then, 8 mg/kg anti-PD1 antibody was injected 3 days later. The tumor growth was significantly inhibited after administration of cisplatin plus anti-PD1 antibody, while cisplatin or PD1 antibody monotherapy showed a moderate anti-tumor efficacy (Figures 5A,B, Supplementary Figures S3A,B). On the other hand, ferrostatin-1 hampered the synergistic anti-tumor effects of combination of cisplatin and PD1 antibody (Figures 5A,B, Supplementary Figures S3A,B). After dissection of tumors, tumor cells were isolated, and the ferroptosis status was detected. Flow cytometry showed that cisplatin treatment could effectively induce ferroptosis of tumor cells and ferrostatin-1 would rescue the cisplatin-induced ferroptosis (Figures 5C–F). The IHC staining further confirmed that cisplatin treatment increased Ly6 and ICAM1 levels, which presented with N1 neutrophils in the tumors (Figure 5G). Additionally, the T-bet level, indicating Th1 cells, was also elevated in the cisplatin treatment group (Figure 5G). However, the number of both neutrophils and Th1 cells decreased in the cisplatin plus ferrostatin-1 treatment group compared with the cisplatin treatment group (Figure 5G). Collectively, our data suggested that cisplatin-mediated ferroptosis of lung cancer cells could increase neutrophil and Th1 cell enrichment in tumor tissues, thereby promoting the synergistic anti-tumor efficacy of ICI therapy.

### 3.6 Ferroptosis Induced the Sensitivity of Epidermal Growth Factor Receptor-Mutant Non–Small Cell Lung Cancer to Immunotherapy

**FIGURE 6:**
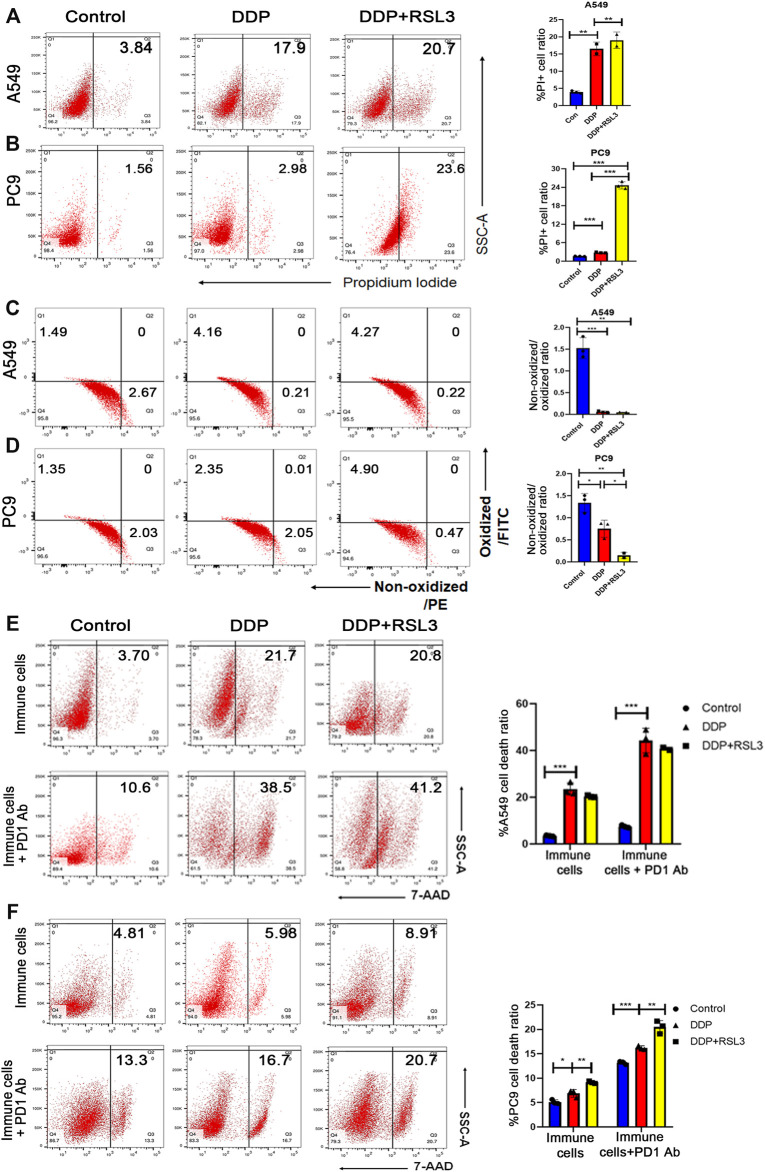
Ferroptosis induced the sensitivity of EGFR-mutant NSCLC to immunotherapy. **(A)** and **(B)** PI staining indicates that more A549 cells die after DDP treatment than PC9 cells. However, DDP plus RSL3 significantly increased cell death ratio in PC9 cells, while RSL3 did not induce a much higher level of cell death in A549 cells. **(C)** and **(D)** Lipid peroxidation assays indicated that A549 cells showed more lipid peroxidation after DDP treatment than PC9 cells. DDP plus RSL3 results in more lipid peroxidation in PC9 cells while RSL3 did not induce much more lipid peroxidation in A549 cells. **(E)** Representative flow cytometry images of A549 cell death ratio in the co-culture system with immune cells. An addition of RSL3 to DDP did not significantly increase cell death level in the co-culture before and after PD1 administration. **(F)** Representative flow cytometry images of PC9 cell death ratio in the co-culture system with immune cells. An addition of RSL3 to DDP significantly increases cell death level in the co-culture before and after PD1 administration. Bar graphs represent the mean ± SD of indicated samples. ∗*p* < 0.05, ∗∗*p* < 0.01, ∗∗∗*p* < 0.001.

The outcomes of clinical trials in the EGFR-mutant NSCLC subgroup showed a generally low response to ICI therapy or ICI therapy plus chemotherapy (Lee et al., 2017). Therefore, there is an urgent need to explore the mechanism of ICI resistance in EGFR-mutant NSCLC. Importantly, neutrophil level in EGFR-mutant NSCLC in TCGA cohort was significantly lower than that in EGFR wild-type cohort, indicating that status of neutrophils could be associated with ICI resistance of EGFR mutant tumors (Supplementary Figure S4). Then, A549 cells (EGFR wild-type) and PC9 cells (EGFR-mutant-type with exon 19del) were used to determine whether EGFR mutation could affect cisplatin-induced ferroptosis and the following status of neutrophils. Cisplatin treatment resulted in a higher cell death in A549 cells than in PC9 cells (Figures 6A,B). Simultaneously, a noticeably higher lipid peroxidation was observed in A549 cells upon cisplatin treatment than in PC9 cells after cisplatin treatment (Figures 5C,D), suggesting a stronger ferroptosis of A549 cells upon cisplatin treatment. On the other hand, RSL3 (a ferroptosis inducer) could further increase ferroptosis of PC9 cells, while it could not additionally increase ferroptosis of A549 cells (Figures 5A,C). These results suggested that the cisplatin alone could not effectively induce ferroptosis, whereas its combination with a cisplatin ferroptosis inducer could lead to a high level of ferroptosis in lung cancer cells with EGFR mutation.

Therefore, we hypothesized that EGFR-mutant NSCLC would take advantage from ICI therapy when ferroptosis was induced in tumor tissues. Thus, we pretreated NSCLC cells with cisplatin or cisplatin plus RSL3 to enhance ferroptosis and then co-cultured them with immune cells isolated from the peripheral blood to polarize neutrophils. Subsequently, untreated tumor cells were co-cultured with these neutrophils plus anti-PD1 antibody. We found that immune cells co-cultured with A549 cells pretreated by cisplatin plus RSL3 had a similar efficacy in killing tumor cells to those co-cultured with A549 cells pretreated by cisplatin alone (Figure 6E). However, immune cells co-cultured with PC9 cells pretreated by cisplatin plus RSL3 had a higher efficacy in killing tumor cells than those co-cultured with PC9 cells pretreated by cisplatin alone (Figure 6F). More importantly, when anti-PD1 antibody was added into the co-culture system, immune cells even showed a higher cytotoxic effect on PC9 cells (Figure 6F).

In particular, a few studies (Ma et al., 2021; Xu et al., 2021) have shown that ferroptosis, as a double-edged sword, can dampen anti-tumor immunity by inducing cell death of CD8 T cells. We next wanted to explore the susceptibility of different cells to ferroptosis. We treated A549 cells and different types of immune cells with 5 μM RSL3 for 12 h. The results suggest that tumor cells, compared with immune cells, are more prone to lipid peroxidation (Supplementary Figures S5A,B) and cell death (Supplementary Figures S5C,D), indicating that tumor cells are the most sensitive to ferroptosis inducers.

Taken together, these results suggested that a ferroptosis inducer could enhance cisplatin-mediated ferroptosis of EGFR-MT NSCLC cells, thereby promoting the anti-tumor immune response of ICI therapy.

## 4 Discussion

The development of ICIs is revolutionizing cancer treatment (McLaughlin et al., 2020). In most types of cancer, only a minority of patients currently benefit from ICI therapy (Spahn et al., 2020). Intrinsic and acquired resistance to ICIs has stimulated scholars to concentrate on new combination therapies to increase response rates, the depth of remission, and the durability of benefit.

The combination with chemotherapy has proved to be an effective method to enhance immune response of ICIs (Galluzzi et al., 2020). Among the first-line chemo-drugs, cisplatin can kill cancer cells and trigger the release of pro-inflammatory mediators to increase tumor-infiltrating immune cells (Grabosch et al., 2019). The present study went one step forward by describing how cisplatin could induce tumor cell ferroptosis to boost immune response and promote the therapeutic effects of ICIs. According to our study, cisplatin could promote ferroptosis of tumor cells and then enhance the N1 neutrophil polarization. The N1-polarized neutrophils release chemokines to facilitate T-cell infiltration and promote Th1 differentiation in the TME. As the most direct effector of anti-tumor immunity, CD8 T cells are activated under the interaction of N1-type neutrophils or Th1 cells, and their infiltration and proliferation abilities are significantly enhanced.

Ferroptosis is an immunogenic cell death (ICD) (Tang et al., 2020), which is driven by iron accumulation and unrestricted lipid peroxidation (Bertrand, 2017). The interaction between ferroptosis and immunity has been a hot topic since its discovery in 2012. Herein, we found that cisplatin could induce ferroptosis of tumor cells. Ferroptosis could release various damage-associated molecular patterns (DAMPs) or lipid metabolites to regulate the cellular immune response. Our study revealed that cisplatin-mediated cancer cell ferroptosis stimulated cancer cells to release higher levels of cytokines and chemokines for neutrophil recruitment and activation. Additionally, the N1-polarized neutrophils activated by cancer cell ferroptosis showed a stronger anti-tumor potential, which is consistent with the result of a previous study, in which ferroptosis-mediated DAMPs could bind to specific receptors to activate inflammation and start immune cell recruitment of neutrophils and monocytes to initiate a pro-inflammatory TME (Gong et al., 2020).

Studies have shown that radiation therapy (Lhuillier et al., 2019), chemotherapy drugs (Chen and Emens, 2013), and targeted oncogene pathway inhibitors (Sun et al., 2018) could achieve this effect through immunogenic cell death and altered tumor cell biology. In addition to directly promoting the anti-tumor pro-inflammatory phenotype of neutrophils, our findings suggested that the ferroptosis-activated neutrophils could further promote T-cell infiltration *via* secreting CXCL9, CXCL10, and CXCL11 and enhance Th1 differentiation *via* secreting IL12A and IL12B in the TME. Therefore, a “cold” TME would become a “hot” one under the interventions. Our findings further supported that the innate immunity might be a trigger for anti-tumor immunity of the TME. Driver mutations in NSCLC were shown as “cold tumors” (Skoulidis and Heymach, 2019). Therefore, there is an urgent need to develop strategies to turn driver-mutant NSCLC into “hot tumors” *via* increasing the infiltration of tumor antigen-reactive T cells for further effective immunotherapy. The present study revealed that amplification of ferroptosis of NSCLC cells could also enhance the immune response *via* a similar mechanism, suggesting a promising strategy to elevate the sensitivity of ICI therapy to driver mutations in NSCLC.

In this study, *in vitro* experiments showed that the ferroptosis inducer RSL3 mainly caused ferroptosis in tumor cells. This indicated that among various cell subtypes in the TME, tumor cells are more susceptible to ferroptosis. However, we observed that the cell death of lymphocytes also increased after the action of RSL3, although not as much as the increase in the proportion of tumor cells. Tumor cells and T cells have great differences in energy metabolism, redox levels, and maintenance of cell survival. Therefore, how to induce ferroptosis in tumor cells specifically and enhance the anti-tumor immune response of immune cells need further exploration.

In summary, the present study revealed that ferroptosis is a major form of cisplatin-mediated immunogenic cell death, playing a key role in amplifying the immune response of ICI therapy. Moreover, boosting ferroptosis would be a potential method to sensitize driver mutations in NSCLC to ICI therapy.

## Data Availability

The original contributions presented in the study are included in the article/Supplementary Material, further inquiries can be directed to the corresponding authors.

## Glossary

ALK: anaplastic lymphoma kinase
ATCC: American type culture collection
BLCA: bladder urothelial carcinoma
BRCA: breast invasive carcinoma
CCL2: C-C motif chemokine ligand 2
CCL3: C-C motif chemokine ligand 3
CCR5: C-C motif chemokine receptor 5
CXCL1: C-X-C motif chemokine ligand 1
CXCL2: C-X-C motif chemokine ligand 2
CXCL10: C-X-C motif chemokine ligand 10
CXCL11: C-X-C motif chemokine ligand 11
DAMPs: damage-associated molecular patterns
DDP: cisplatin
DEG: differentially expressed gene
EGFR: epidermal growth factor receptor
ELANE: elastase
ELISA: enzyme-linked immunosorbent assay
ESCA: esophageal carcinoma
FDR: false discovery rate
GDC: Genomic Data Commons
GO: gene ontology
GSEA: gene set enrichment analysis
GZMB: granzyme B
HNSCC: head and neck squamous cell carcinoma
ICD: immunogenic cell death
ICIs: immune checkpoint inhibitors
IFNA2: interferon alpha 2
IFNB: interferon beta
IFN-γ: interferon gamma
IHC: immunohistochemistry
IL2: interleukin 2
IL12: interleukin-12
KEGG: Kyoto Encyclopedia of Genes and Genomes
LLC: Lewis lung carcinoma
LUAD: lung adenocarcinoma
LUSC: lung squamous cell carcinoma
MACS: magnetic activated cell sorting
NSCLC: non–small cell lung cancer
PBMCs: peripheral blood mononuclear cells
PD-1: programmed cell death protein 1
RT-qPCR: quantitative reverse transcription polymerase chain reaction
ssGSEA: single sample gene set enrichment analysis
TCGA: The Cancer Genome Atlas
TIME: tumor immune microenvironment
TME: tumor microenvironment
TNF-α: tumor necrosis factor-α
TNFB: tumor necrosis factor B
