# Supplementary material for: Cisplatin Promotes the Efficacy of Immune Checkpoint Inhibitor Therapy by Inducing Ferroptosis and Activating Neutrophils
Source: Front Pharmacol. 2022 Jun 13;13:870178. doi: 10.3389/fphar.2022.870178 (PMC9240830; doi:10.3389/fphar.2022.870178)
Supplement: Supplementary file 4 [file DataSheet1.pdf]

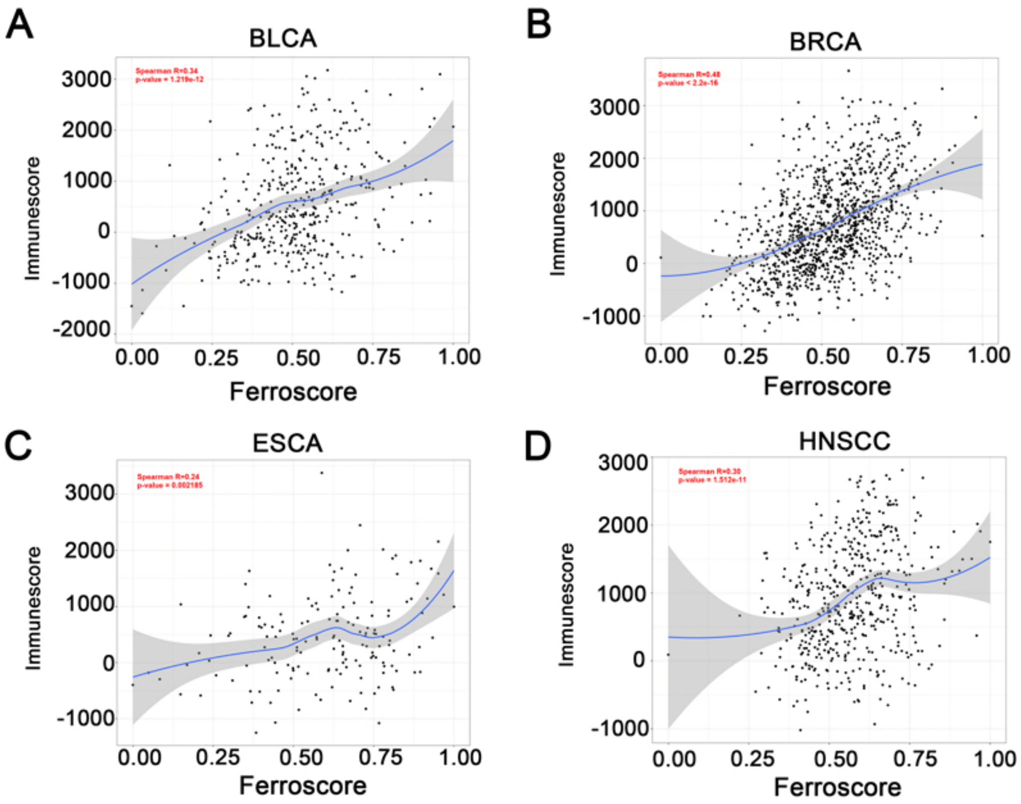

Supplementary figure1. Cisplatin promoted tumor ferroptosis that was correlated with immune score. A -D: The ferroscore correlated with immunescore in BLCA (A), BRCA (B), ESCA(C), HNSCC(D) cohorts of TCGA database.

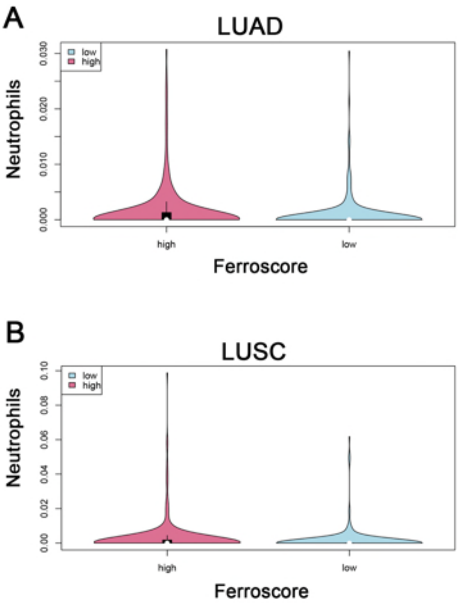

Supplementary figure2. The Ferroscore was correlated with neutrophils.
A-B. The xCELL algorithm showed the ferroscore-H group was correlated with a higher level of neutrophils in both LUAD(A) and LUSC(B) cohort.

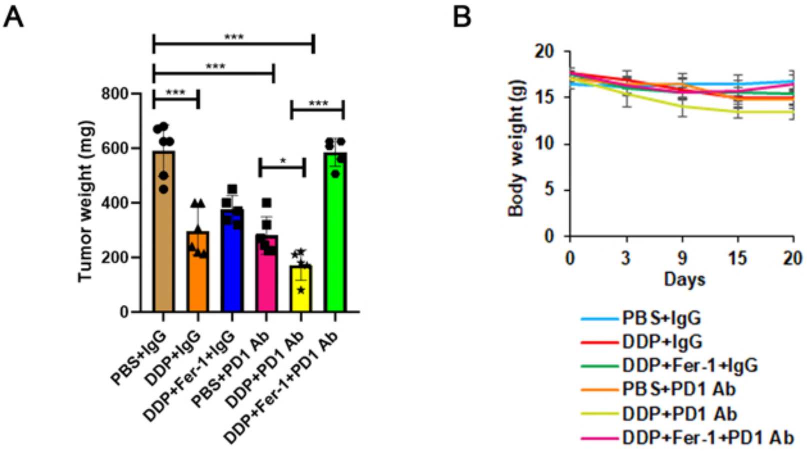

Supplementary figure3. Cisplatin-induced ferroptosis promoted efficacy of ICI therapy in vivo. A and B: Tumor weight (A) and body weight (B) of Lewis lung cancer cells (LLC) bearing C57 mice.

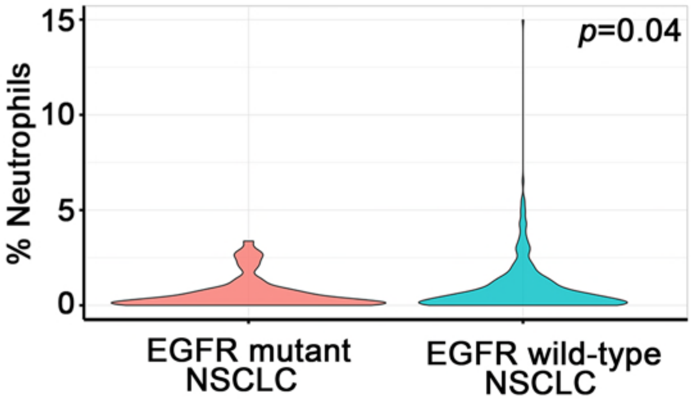

Supplementary figure4. The neutrophil level in EGFR-mutant and wild type NSCLC in TCGA cohort.

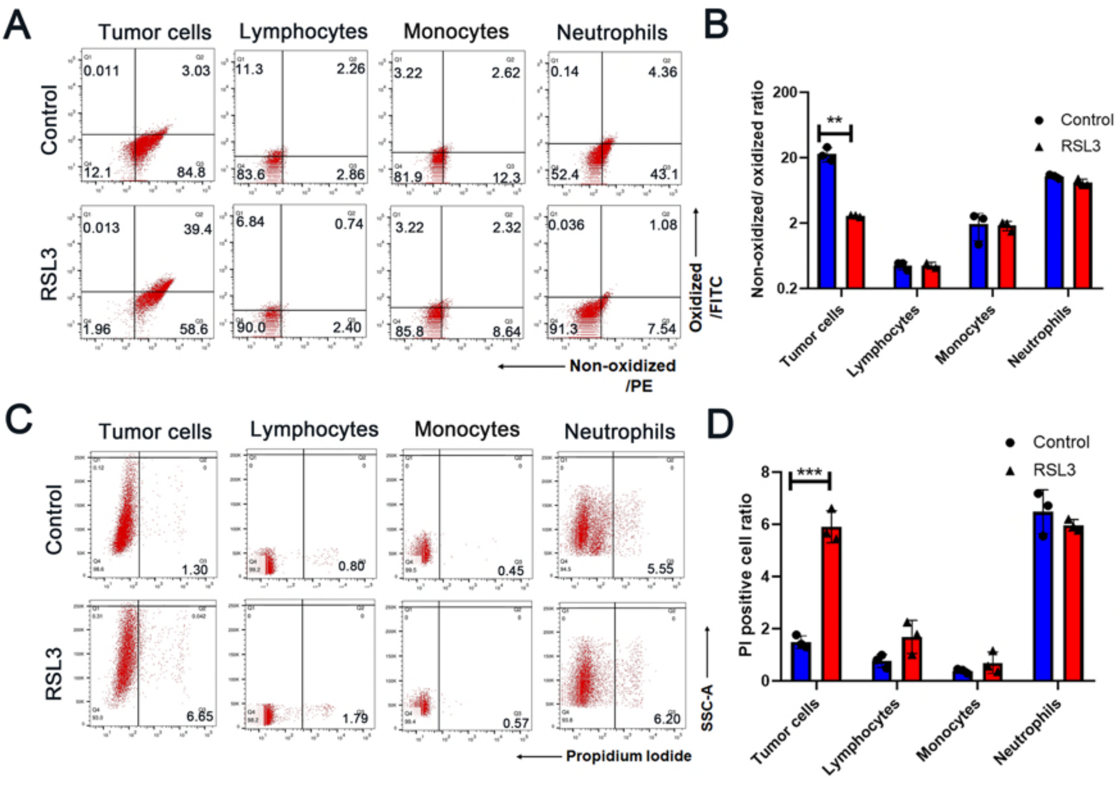

Supplementary figure5. Tumor cells are more susceptible to ferroptosis than immune cells. (A) Tumor cells showed higher level of lipid peroxidation than lymphocytes, monocytes and neutrophils after RSL3 treatment. (B) Quantification of data in (A).(C)The cell death ratio of tumor cells is much higher than lymphocytes, monocytes and neutrophils after RSL3 treatment. (D) Quantification of data in (C).$^{**}p<0.01$, $^{***}p<0.001$.
